# Characterization of a New Epidemic Necrotic Pyoderma in Fur Animals and Its Association with *Arcanobacterium phocae* Infection

**DOI:** 10.1371/journal.pone.0110210

**Published:** 2014-10-10

**Authors:** Heli Nordgren, Kirsi Aaltonen, Tarja Sironen, Paula M. Kinnunen, Ilkka Kivistö, Mirja Raunio-Saarnisto, Anna-Maria Moisander-Jylhä, Johanna Korpela, Ulla-Maija Kokkonen, Udo Hetzel, Antti Sukura, Olli Vapalahti

**Affiliations:** 1 Production Animal Section, Finnish Food Safety Authority (Evira), Seinäjoki, Finland; 2 Department of Veterinary Biosciences, Faculty of Veterinary Medicine, University of Helsinki, Helsinki, Finland; 3 Department of Virology, Haartman institute, University of Helsinki, Helsinki, Finland; 4 Seinäjoki Laboratory Section, Finnish Food Safety Authority (Evira), Seinäjoki, Finland; 5 Finnish Fur Breeders Association (FFBA), Vantaa and Vaasa, Finland; 6 Section for Poultry, Wild and Laboratory Animals, Finnish Food Safety Authority (Evira), Helsinki, Finland; 7 Department of Virology and Immunology, HUSLAB, Hospital district of Helsinki and Uusimaa, Helsinki, Finland; The University of Melbourne, Australia

## Abstract

A new type of pyoderma was detected in Finnish fur animals in 2007. The disease continues to spread within and between farms, with severe and potentially fatal symptoms. It compromises animal welfare and causes considerable economic losses to farmers. A case-control study was performed in 2010–2011 to describe the entity and to identify the causative agent. Altogether 99 fur animals were necropsied followed by pathological and microbiological examination. The data indicated that the disease clinically manifests in mink (*Neovison vison*) by necrotic dermatitis of the feet and facial skin. In finnraccoons (*Nyctereutes procyonoides*), it causes painful abscesses in the paws. Foxes (*Vulpes lagopus*) are affected by severe conjunctivitis and the infection rapidly spreads to the eyelids and facial skin. A common finding at necropsy was necrotic pyoderma. Microbiological analysis revealed the presence of a number of potential causative agents, including a novel *Streptococcus* sp. The common finding from all diseased animals of all species was *Arcanobacterium phocae*. This bacterium has previously been isolated from marine mammals with skin lesions but this is the first report of *A. phocae* isolated in fur animals with pyoderma. The results obtained from this study implicate *A. phocae* as a potential causative pathogen of fur animal epidemic necrotic pyoderma (FENP) and support observations that the epidemic may have originated in a species -shift of the causative agent from marine mammals. The variable disease pattern and the presence of other infectious agents (in particular the novel *Streptococcus* sp.) suggest a multifactorial etiology for FENP, and further studies are needed to determine the environmental, immunological and infectious factors contributing to the disease.

## Introduction

In 2007, Finnish fur farmers and the industry were affected by a new type of disease pattern in fur animals characterized by severe suppurative skin inflammation (pyoderma), affecting all major fur animal species in the country: captive mink (*Neovison vison*), foxes (*Vulpes lagopus*) and finnraccoons (*Nyctereutes procyonoides*, a raccoon dog bred in Finland for the fur industry). The disease manifests slightly differently in each species, but pyoderma is the common feature. The disease causes significant economic losses for the fur industry and affects animal wellbeing. Similar signs were documented in farmed mink in Canada in 1996 and also in USA in 1970 [1]. According to the Canadian farmers, the occurrence of the clinical signs was linked to the feeding of the mink with byproducts of seal. The Canadian study suggested that the cause of pododermatitis in mink was an unidentified infectious agent with secondary bacteriologic pyoderma. Similar signs have, to the best of our knowledge, never been described in foxes and finnraccoons. The disease pattern has been detected in fur animals in Denmark, Poland, The Netherlands, Spain, Iceland, and most recently in Sweden and Norway (personal communication from veterinarians treating fur animals, Nordic Association of Agricultural Scientists, NJF, Fur animal veterinarian meeting, Levi, Finland, 2014).

In mink, bacterial dermatitis is a relatively common finding and often associated with biting wounds. Only a few young individuals are usually affected [2]. The most common bacteria found on infected skin are *Staphylococcus* spp. and *Streptococcus* spp. These infections are less common in finnraccoons and foxes. The new deep pyoderma differs from previous disease patterns by spreading within the farm, and in the worst case affecting the entire pack.

In 1997, a novel bacterium, *Arcanobacterium phocae*, was isolated from gray seals (*Halichoerus grypus*) and common seals (*Phoca vitulina*) around Scotland, UK [3]. Skin lesions associated with *A. phocae* were first described in a study analyzing samples gathered between 1994 and 2000; *A. phocae* isolates were recovered from 141 marine mammals, stranded along the central California coast (USA). *A. phocae* was cultured from 66 California sea lions (*Zalophus californianus*), 50 Pacific harbor seals (*Phoca vitulina richardii*), 19 northern elephant seals (*Mirounga angustirostris*), five southern sea otters (*Enhydra lutris nereis*), and one common dolphin (*Delphinus delphis*). In marine mammals, *A. phocae* isolates are most commonly obtained from superficial pyogenic infections such as abscesses, wounds and exudates. However, some cases with deep-seated and systematic infections have also been documented. Many of the infections are associated with bite and bullet wounds or other kinds of traumatic skin injury. *A. phocae* isolates from marine mammals are often present in mixed bacterial infections [4].

*Arcanobacterium phocae* is a Gram-positive coccobacillus that occasionally morphs into a short rod. The bacteria are non-motile and beta-hemolytic on blood agar. *A. phocae* is catalase positive and oxidase negative and in the CAMP reaction test, a reverse CAMP reaction with *Staphylococcus aureus* and a positive CAMP reaction with *Rhodococcus equi* are typical. It has also been found to be susceptible to all tested antibiotics including aminoglycosides, β-lactams, bacteriostatic and bacterisidal antibiotics, fluoroquinolones, macrolides, rifamycins, and polyketides [4].

*A. phocae* belongs to the genus *Arcanobacterium* first described by Collins *et al.* in 1982 [5]. This genus is currently under taxonomic revision [6]: the genus *Arcanobacterium sensu stricto* has been suggested to contain the species *A. haemolyticum*, *A. phocae*, *A. pluranimalium*, and *A. hippocoleae*. In addition, the recently discovered *A. canis* [7] and *A. phocisimile* [8] have been suggested to belong to this genus. Five previous members of the genus *Arcanobacterium* have been proposed to form a new genus, *Trueperella* [6]: *T. bernardiae*, *T. pyogenes*, *T. bialowiezense*, *T. bonasi*, and *T. abortisuis*. These bacteria may be recovered from various organs such as the respiratory and digestive tracts, abscesses and systemic infections. *Arcanobacterium* spp. and *Trueperella* spp. have also been reported from other infections and colonization of both healthy and diseased humans and animals [3], [9]–[11].

In 2010, the Finnish Fur Breeders association (FFBA), Finnish Food Safety Authority Evira and University of Helsinki (UH) started a collaborative project to describe the clinical signs, gross and histological lesions, epidemiological aspects, as well as the etiological agent(s) of the disease. Here, we describe the manifestation and pathology of the fur animal epidemic necrotic pyoderma (FENP) and provide microbiological evidence for *Arcanobacterium phocae* as its candidate etiological agent.

## Materials and Methods

### Ethics statement

The Animals investigated here were culled for pelting as part of normal procedures in fur animal husbandry, and thus no ethical permission was required in any of the organizations or institutions involved. The carcasses of all included individuals (99) were obtained as donations from 13 fur animal farms. No animals were culled for research purposes. The mink were euthanized on the farm using CO or CO_2_ gas, and foxes and finnraccoons using electricity by the methods described in legislation concerning culling of the animals ((EU) N: o 1099/2009). The animal welfare is secured on the farms by a Certification Program, created and managed by the Finnish Fur Breeders Association. The certification program means continuous development in the farms, strict monitoring and documentation of all activities. The samples used in this study came from certified farms, and the farms voluntarily sent the samples to Evira.

### Sampling

Between November 2010 and January 2011, 21 mink, 19 foxes and 21 finnraccoons exhibiting typical signs were gathered from 10 farms with a history of disease. Furthermore, 11 mink and finnraccoons and 12 foxes were taken from three farms where the disease had never been detected, and a further four clinically healthy mink were collected from two diseased farms (altogether 99 animals). A complete necropsy with histological and microbiological examination was performed on all animals. The animals were gathered at pelting time, when mostly young animals (average seven months) are kept on the farms with equal numbers of both sexes. The animals used in the study were not treated with any medication.

The 21 diseased mink were collected from two (17+4) farms. Both sexes were represented (nine females and 12 males). Sampled mink were under one year of age. Both diseased and healthy mink had been vaccinated against virus enteritis caused by mink enteritis parvovirus (MEV), botulism caused by *Clostridium botulinum* and hemorrhagic pneumonia caused by *Pseudomonas aeruginosa*.

The 19 diseased foxes were collected from three farms (11+5+3), and healthy controls (12) from one farm. Most of the foxes were less than one year old, but some lacked anamnestic information. Both sexes were represented but diseased females predominated (16) and healthy controls were all male. None of the farms had vaccinated the foxes against parvovirus.

The 21 diseased finnraccoons originated from five farms (2+12+4+2+1) and healthy controls (11) from one farm. Most of the diseased finnraccoons were female (13) and most of the healthy controls were male (7). All these finnraccoon farms vaccinated the animals against parvovirus with the mink enteritis vaccine.

### Gross pathology

The gross lesions were described by the location, width and severity (mild, moderate, severe) of the lesion and the duration (acute, chronic) of the process. Any lesions in the internal organs were also recorded.

### Histopathology

Samples of brain, heart, lung, trachea, spleen, liver, kidney, bladder, duodenum, jejunum, ileum, colon, local lymph nodes, and skin (mink, finnraccoon) and eyes (fox) were fixed in 10% phosphate buffered formalin for at least 24 h, embedded in paraffin and sectioned at 4 µm. All tissues were stained with hematoxylin and eosin (H&E), and selected tissues with special stains (Gomori Methenamine Silver (GMS), Zieh-Neelsen (ZN), and Warthin Starry silver stain (WS)).

### Bacteriological studies

Bacteriological investigations were performed in the Seinäjoki unit of Evira. Samples of brain, heart, lung, spleen, liver, kidney, duodenum, jejunum, ileum, colon, local lymph nodes, and skin (mink, finnraccoon) and eyes (fox) were cultured on blood agar plates containing 5% defibrinated bovine blood and incubated aerobically at 37°C for 24–48 hours. Skin and eye samples were also cultured anaerobically at 37°C for 4–7 days. Earlier investigations found no yeast, fungi, *Salmonella*, *Campylobacter* spp., other anaerobic pathogens or mycoplasma in the diseased animals, so these tests were excluded from this study. Bacterial species were confirmed either by biochemical methods or 16S RNA PCR and sequencing. Three isolates were tested for antibiotic susceptibility with VetMIC-panels for Gram-positive bacteria (VetMIC GP mo) and for small animals (VetMIC smådjur) (The National Veterinary Institute, SVA, Sweden).

### PCR detection of *Arcanobacterium phocae*

Due to the relatively low sensitivity of bacterial cultures and the nature of the sample material, with the potential loss of bacterial cultivability, a PCR -method was developed for the detection of *A. phocae*. Tissue biopsy samples (third eyelid and tissue samples from affected paws or face) were homogenized by mortar and pestle or by cutting with scalpel. DNA was extracted from these homogenates as well as from the eye swabs using a DNA blood and tissue kit (Qiagen) according to the manufacturer's instructions, with tissue lysis overnight. PCR primers Forw-5′-TGGCATGCTGTTGGGGTGT-3′ and Rev-5′-TCGGCTCCGTATGCCAAGGC-3′ were designed to amplify a 182-nt product from the 16S–23S intergenic region. Real-time PCR was performed using the MAXIMA SYBR Green kit (Thermo Fisher Scientific) in a StrataGene Mx3005p thermocycler. The program contained a preliminary denaturation step of 10 minutes at 95°C followed by 40 cycles of denaturation at 95°C for 15 s, annealing at 65°C for 60 s and extension at 72°C for 60 s, with measurement after the annealing step. The specificity of the products was checked by running a melting curve analysis and by direct sequencing of the products. The analytical sensitivity of the PCR assay was estimated by a dilution series of DNA extracted from a pure culture of *A. phocae*, and was determined to be one bacterial genome per reaction. The sensitivity in sample matrix was assayed by spiking negative tissue samples with the same extraction, and was shown to be ten bacterial copies per reaction. The copy number was estimated using the genomic size of the closest known relative, *Arcanobacterium haemolyticum* and a copy number counter (Thermo Fisher Scientific). The analytical specificity of the assay was estimated using DNA extracted from *Escherichia coli*, *Staphylococcus pseudintermedius*, *Streptococcus halichoeri*, *Streptococcus canis*, *Streptococcus pyogenes*, *Arcanobacterium haemolyticum* and the novel *Streptococcus* found in this study. These all remained negative, except in extremely high concentrations of pure bacterial cultures. This led to the definition of a diagnostic cut-off for the cycle threshold value (Ct) set to 31 approximately corresponding to 50 copies (Table S1). For samples above this cut-off, but with the correct melting temperature, sequencing was performed to confirm the specificity of each positive result.

### Virological studies

Serum and tissue samples (lung, trachea, bladder and rectum) were collected and sent to the Helsinki unit of Evira for virological studies. Canine distemper virus (CDV) was tested from the lung, trachea, and bladder samples, and mink enteritis parvovirus (MEV) from the rectum samples, both by PCR modified from [12] and [13], respectively. Antibodies against CDV and MEV were measured from the serum collected from the necropsied animals. The serum neutralization test was used for the detection of antibodies to CDV [14], and the hemagglutination inhibition test for antibodies to MEV [15]. Pieces of the affected skin and eyes were frozen in Universal Transport Medium (UTM) -tubes (Copan, USA) at −70°C and sent to the University of Helsinki to further investigations. From these samples, positivity for herpesvirus DNA was tested with PCR [16]. All mink were tested against plasmacytosis (Aleutian Mink Disease virus) in the laboratory of the Finnish Fur Breeders Association in Vaasa with an ELISA-test [17].

### Virus isolation trials

Tissue samples from infected animals were homogenized by manual grinding with mortars and pestles over dry ice. Dulbecco's modified phosphate buffered saline solution with 0.2% bovine serum albumin, 10 U/ml penicillin, 0.1 mg/ml streptomycin, and 0.25 µg/ml amphotericin B (Fungizone, Invitrogen) was used in the homogenizing with 700 µl of D-PBS added to approximately 10–50 mg of tissue. Swabs taken from infected eyes were vortexed and 100 µl of the medium was diluted with 400 µl of D-PBS. Samples of the homogenate and transport medium were collected for nucleic acid extraction and electron microscopy (EM), for which samples were negatively stained with uranyl acetate and the potential presence of viruses studied using a JEOL 1400 transmission electron microscope at 80 kV.

A preliminary isolation test was performed using Madin-Darby canine kidney epithelial (MDCK, ATCC CCL-34), Mink Lung epithelial (Mv1Lu, ATCC CCL-64), canine tumor fibroblast (A-72, ATCC CRL-1542), and feline kidney epithelial (CRFK, ATCC CCL-94) cell lines. Based upon the cell type and species match, and tolerance of long growth times, MDCK and Mv1Lu cell lines were chosen for the main experiment. The MDCK cells were propagated in Eagle's minimum essential medium (MEM) with 10% fetal bovine serum (FBS), 0.3 mg/ml glutamine, 10 U/ml penicillin, and 0.1 mg/ml streptomycin and the Mv1Lu cells in Dulbecco's modified minimum essential medium (D-MEM) with added glucose and 7% fetal bovine serum (FBS), 0.3 mg/ml glutamine, 10 U/ml penicillin, and 0.1 mg/ml streptomycin. The cells were incubated at 37°C with 5% CO_2_, and were grown to approximately 75% confluence on six well plates before infection. After washing the cells with PBS, 300 µl of homogenate was pipetted onto them and incubated for 1 hour with gentle shaking every 15 minutes. One milliliter of the cell-specific growth medium with only 2% FBS was added onto the cells and they were incubated over night, after which another milliliter of this medium was added.

Cell cultures were followed for signs of cytopathic effect (CPE) daily, and samples of the medium and cells were collected when CPE was detected. The culture medium was changed once a week and the cells were allowed to grow for approximately 3 weeks. Samples of the medium containing cells were also collected when the medium was changed. Selected samples showing possible CPE were assessed by EM as described earlier in this section.

## Results

### Clinical signs of fur animal epidemic necrotic pyoderma

The disease was found in all three species of fur animals: mink (*Neovison vison*), foxes (*Vulpes lagopus*), and finnraccoons (*Nyctereutes procyonoides*). However the clinical manifestation differed in each of them. Commonly observed signs in affected mink included skin inflammation on the paws and the facial skin. Any paw could be affected, but inflammation was usually first noted on the front paws. Lesions on the head were usually around the eyes, ears, or nose. In foxes, the first clinical sign detected was anorexia, followed by serous discharge from the eyes, which rapidly turned purulent. In some cases, the suppurative inflammation spread to the eyelids and the facial skin. In finnraccoons, the infection was usually limited to the paws. Paws were swollen, and in severe cases deep abscesses formed and the infection could proceed proximally along the limbs. Poor appetite was observed in prolonged cases.

### Gross lesions in the study animals

**Figure 1:**
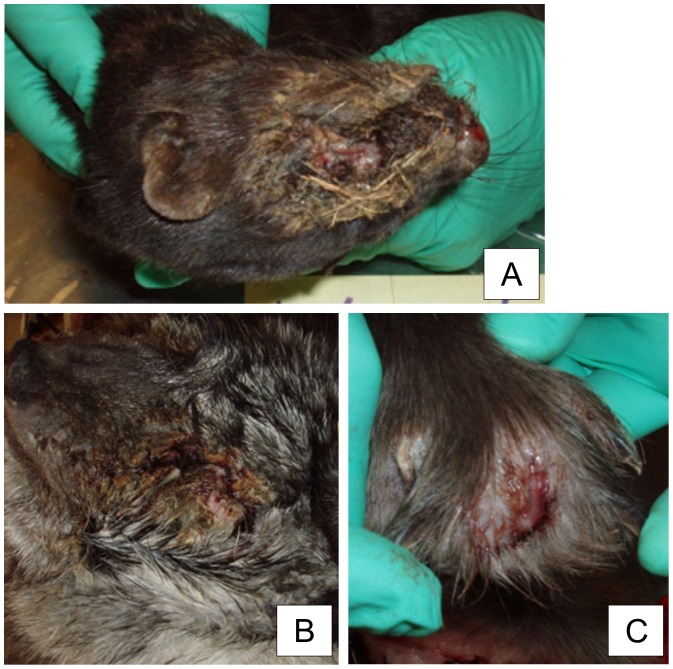
Macroscopic changes in FENP in mink, foxes and finnraccoons. A typical macroscopic finding in a mink with FENP is severe necrotic pyoderma with crust formation around the eyes and nose. Some bedding material is detached in the exudate (A). A typical lesion in foxes with FENP is observed around the eyes. The eyelids clot together due to the purulent inflammation (B). Finnraccoons with FENP have painful abscesses between the toes (C).

The mink typically (15/21) had chronic severe necrotic pyoderma in the facial skin. Crust formation was detected with brownish exudate and attached bedding material (Fig. 1a). Lesions ranged from 2 cm×2 cm to 8 cm×8 cm and often covered the nose, eyes, and ears. Beneath the crust tissue, necrosis and hyperemia were observed. Some mink (3) had an alopecic ring around the lesion. Similar but smaller-sized lesions were seen on the legs in five out of 21 necropsied mink (Table 1). One mink had lesions on a hind leg, and others on their front legs (4/21). Lesions were observed on the footpads, nail beds, or dorsal skin of the paw. Two mink had a nail missing and the inflammation had spread to the bone structures. One mink only had alopecia in the dorsal skin of the paw, with no ulcer or crust formation. The exact progression of the disease could not be recorded, as all samples represented chronic inflammation. Splenomegaly was seen in 10/21 of the diseased mink, and 7/21 had swollen and enlarged local axillary or popliteal lymph nodes (Table 1). One mink had fatty liver, considered as an incidental finding, and two were extremely cachectic. Congestion in internal organs was seen in 4/21 animals.

**Table 1:** Gross pathology of the study animals.

Macroscopic change	Mink (n = 21)	Foxes (n = 19)	Finnraccoons (n = 21)
**Enlarged local lymph node**	5 (22%)	3 (16%)	10 (48%)
**Splenomegaly**	10 (48%)	0	10 (48%)
**Pyoderma in facial skin**	15 (71%)	3 (16%)	0
**Pyoderma/abscess in paws**	6 (29%)	0	21 (100%)
**Bilateral conjunctivitis**	0	17 (89%)	0
**Unilateral conjunctivitis**	0	2 (11%)	0
**Purulent discharge from eyes**	0	11 (58%)	0
**Serous discharge from eyes**	0	8 (42%)	0

Samples of foxes (19) represented a variety of the stages of the disease and it was therefore possible to describe the presumptive disease progression. An early sign of the disease was serous discharge from the eyes followed by the third eyelid becoming hyperemic, with vesicle development. The discharge turned purulent as the disease progressed. The purulent inflammation spread from the eye to the eye lids (Fig. 1b) and sometimes to the entire skin of the face. Entropion was noted in the eyelids of 3/19 of the animals (Table 1). Three foxes had enlarged and swollen local lymph nodes, and two had a fatty liver. In the internal organs no other significant findings were recorded.

Diseased finnraccoons had abscesses between the toes (Fig. 1c), 17/21 had lesions on the front paws, and both front and hind paws were affected in 4/21 (Table 1). The abscesses were intact, but one also had fistulae. One control animal had abscesses between the toes. Altogether, 10/21 of the diseased finnraccoons had splenomegaly and 10/21 had enlarged local lymph nodes (axillary and/or popliteal).

### Histopathology

**Figure 2:**
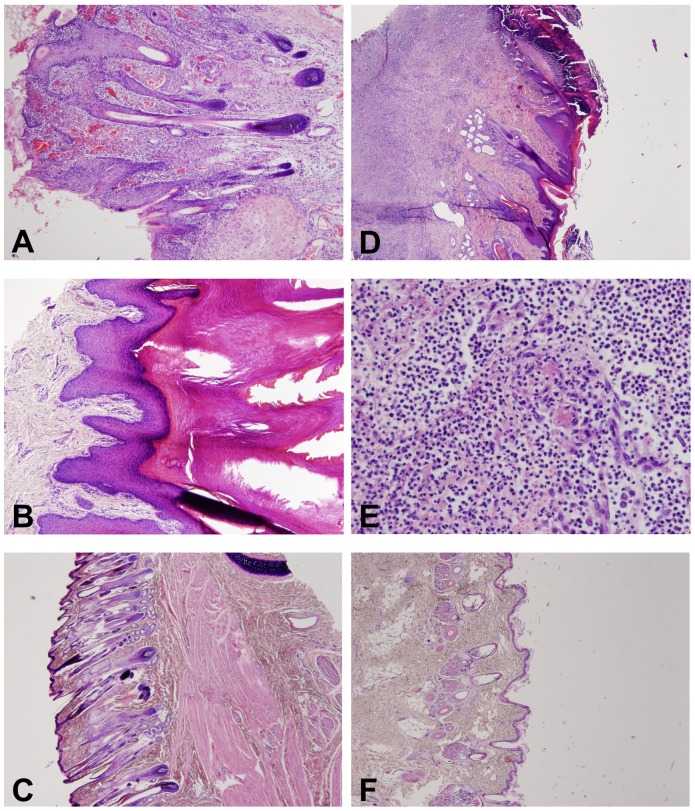
Histopathological findings in FENP in mink and finnraccoons. A facial skin section from mink shows chronic, deep, and diffuse neutrophilic inflammation with hemorrhage, ulceration, and crusting (A). Severe orthokeratotic hyperkeratosis detected in a skin section from a mink foot (B). A sample of a section from the skin of the eyelid of a healthy control mink is shown in (C). A section from a diseased finnraccoon paw with abscess between the toes shows chronic, deep and diffuse neutrophilic inflammation with ulceration and crusting (D). Necropurulent inflammation in the subcutis is demonstrated in (E), and a section from the skin of a paw of a healthy control finnraccoon in (F). All sections are stained with hematoxylin and eosin, and the respective objectives used in A to F were 10×, 10×, 4×, 4×, 40× and, 4×.

Skin samples from 19/21 mink displayed severe chronic necrotic pyoderma with ulceration and crusting, and 11/19 had coccoid bacteria in the epidermal layer (Fig. 2a). Severe necrosis (11/21) and vasculitis (8/21) that had led to thrombosis were detected. The inflammation appeared to spread to hair follicles in 4/21 cases (perifolliculitis/folliculitis). Hyperkeratosis (10/21) and parakeratosis (8/21) were observed in the samples (Fig. 2b). Some vacuolated keratinocytes (4/21) as well as hypergranulosis (4/21) were occasionally seen. Perivascular and periadnexal lympho-plasmacytic inflammation was noted in 9/21 cases. In two samples, eosinophilic material resembling inclusion bodies was detected. Special stains (GMS and ZN) were used, but no evidence of parasites, fungi, or mycobacteria was seen. WS staining revealed no silver stain-positive organisms. The samples represented chronic inflammation, and it is possible that the first insults of infection could no longer be detected in these samples. The healthy controls had no inflammatory changes, but slight hyper- and parakeratosis in the footpads was seen in 2/11 of the healthy controls from the uninfected farm, as well as in two out of the four healthy controls from the diseased farms.

Diseased mink had histologically reactive changes in the spleen (9/12) and in the local lymph nodes (3/12), as well as perivascular or peribronchial lymphocytosis in lung specimens (4). One diseased mink had severe pneumonia with Langerhans cells, syncytia, and alveolar histiocytosis. In the lungs of the healthy controls, varying degrees of perivascular and peribronchial lymphocytosis were detected (4/11). In two healthy individuals, gathered from a diseased farm, perivascular and peribronchial lymphocytosis was observed in the lung samples (2/4). No specific changes were detected in the other internal organs of any of the mink.

The foxes displayed conjunctivitis which spread from the limbus to the corneal center, progressing centrally to an ulcerative keratitis. Occasional findings were made of phtisis, hypopyon, and conjunctival inflammation extending into the retrobulbar/periscleral tissues, and chronic, purulent pustular dermatitis in the eyelids. These findings were not consistent. The inflammation was chronic, lympho-plasmacytic conjunctivitis with mild to moderate, predominantly neutrophilic inflammation. In severe cases, lympho-plasmacytic keratitis and serocellular crusts with coccoid bacteria were present. Hypereosinophilia of basal epithelia, corneal edema and activation of keratocytes was also detected. No specific changes were observed in the internal organs of any of the foxes.

Finnraccoons had varying degrees of chronic deep diffuse neutrophilic inflammation that also involved the subcutis (18/21) (Fig. 2d and e). In many samples, severe furunculosis (13/21) was detected. In 8/21 of the samples there was hyperkeratosis in the epidermis, with ulceration, hemorrhage, and necrosis with coccoid bacteria. Samples were seen with marked eosinophilia in the lesion (5), as well as lympho-plasmacytic inflammation (3), in addition to purulent inflammation. The samples from finnraccoons represented chronic cases. On necropsy, one of the individuals submitted to the study as a healthy control was observed to exhibit the typical lesions of FENP. The histopathological changes were also similar to the changes in the diseased animals. No specific changes were detected in the internal organs of any of the finnraccoons.

### Bacteriology

The main finding in bacteriological cultures was *Arcanobacterium phocae*, identified by sequencing of the 16S RNA gene. It was cultured from a total of 16/21 of the diseased mink skin samples and 6/19 of the diseased fox eye samples. In the fox samples with purulent discharge, *A. phocae* was cultured from 5/11 samples. In the fox samples with serous discharge, *A. phocae* was only cultured in one sample (1/8). *A. phocae* was cultured from 8/21 of diseased finnraccoon samples. Furthermore the finnraccoon submitted to the study as a healthy control, but exhibiting the typical lesions, was positive for *A. phocae*. The difference between detecting *A. phocae* in primarily diseased mink and foxes but not healthy ones was statistically clearly significant (Table 2, Table S1).

**Table 2:** Detection of *Arcanobacterium phocae* and *Streptococcus* spp. in fur animals, and their association to disease signs of FENP.

	PCR positive for *A. phocae*		Positive for *A. phocae* in culture		Positive for *Streptococcus* spp in culture	
		*p*-values*		*p*-values		*p*-values
**Diseased mink**	21 (21)	<0.001	16 (21)	<0.001	16 (21)	<0.001
Healthy mink	1 (11)		0 (11)		0 (11)	
Healthy mink on a diseased farm	3 (4)		0 (4)		0 (4)	
**Diseased finnraccoons**	20 (20)	<0.01	8 (20)	0.068	10 (20)	<0.01
Healthy finnraccoons	6 (11)		1 (11)		0 (11)	
**Diseased foxes**	19 (19)	<0.001	6 (19)	0.036	2 (19)	0.3677
Healthy foxes	0 (12)		0 (12)		0 (12)	
**All diseased animals**	60 (60)	<0.001	30 (60)	<0.001	28 (60)	<0.001
All healthy animals	10 (38)		1 (38)		0 (38)	

The table shows the probabilities of getting a positive result from diseased animals as opposed to healthy animals.

* Calculated from Fisher's exact test.

On blood agar, *A. phocae* grew in 24 hours as very small, pinpoint-like colonies with a strong hemolytic zone around the colonies. A CO_2_-enriched atmosphere or anaerobic conditions did not enhance their growth. They were small, pleomorphic rods and they stained unevenly Gram-positive. They were catalase- positive, oxidase-negative, and they gave a positive CAMP-reaction to *Rhodococcus equi* and *Streptococcus agalactiae* (synergistic hemolysis), and an inverse CAMP -reaction to *Staphylococcus aureus*.

*A. phocae* was most commonly (26/31) isolated as a mixed culture, together with bacteria belonging to the genus *Streptococcus* or *Staphylococcus*. In five diseased cases, however, *A. phocae* was the only isolate (Table 2). All the tested isolates of *A. phocae* were susceptible to all the antibiotics available for treatment.

Some of the *Streptococcus* isolates (28/38) were determined to represent a previously unknown *Streptococcus* based on the 16S RNA gene sequence. The sequence showed the novel *Streptococcus* to be closely related, but not identical, to streptococci of marine origin, such as *Streptococcus halichoeri*. They also grew on blood agar in 24 hours as very small, pinpoint-like colonies, and CO_2_ -enrichment or anaerobic conditions did not enhance their growth. They were Gram-positive cocci, but they stained unevenly. The colonies were nonhemolytic. They were catalase-positive, oxidase-negative, and were categorized as group B by the Lancefield test. In addition, the finding of the cultivable streptococcal species in diseased mink and finnraccoons, but not foxes, was statistically significant (Table 2).

### PCR studies on *A. phocae*

All the diseased animals of all three species were positive for *A. phocae* DNA (Table 2). Furthermore, most of these had relatively high amounts of bacteria as indicated by the Ct-values in real-time PCR. Most of the samples from the healthy controls of mink and foxes were negative, although some weak positive reactions were detected, indicating very low amounts of bacteria (Table 2). The finnraccoons displayed a somewhat different result: six out of the 11 healthy finnraccoons were positive in PCR for *A. phocae*. The bacterial load was rather low in these negative samples, but some of the diseased finnraccoons had comparable, low, DNA levels. However, the statistical difference in detecting *A. phocae* by PCR in all diseased but only 6/11 healthy finnraccoons was clearly significant (p = 0.003, Fisher's exact test) (Table 2). Evidently, the difference between detecting *A. phocae* in primarily diseased mink and foxes but not healthy ones by PCR was also statistically very highly significant (Table 2). When all animals were pooled together, the statistical significance of finding *A. phocae* DNA predominantly in animals with FENP (Table 2) had a p value of 1.8×10∧−16 (Fisher's exact test).

### Virology

Canine distemper virus or antibodies against it were not detected in any species. Herpesviruses were not detected by PCR, virus culture, or EM. All mink were negative in an ELISA-test for AMDV and for MEV antibodies. In foxes and finnraccoons, antibodies against MEV were detected in both diseased and healthy animals. MEV DNA was detected in a rectal sample from one healthy fox.

Virus isolations were attempted from the affected tissue samples and eye swabs, and as a control, from corresponding samples of healthy animals. Occasionally, unspecific CPE was observed, and samples of supernatant as well as original tissues were analyzed by electron microscopy. However, no viral agents were detected.

## Discussion

In this study we described a novel disease, fur animal epidemic necrotic pyoderma (FENP), which appeared in Finland in 2007. The clinical signs appeared to be distinct for each fur animal species: necrotic skin inflammation affecting both the head and paws in mink, severe eye inflammation in foxes that spreads to the eyelids and facial skin, and abscesses in the paws of finnraccoons. The disease mostly affected young animals of both sexes. It appeared to have spread within and between farms, and was evidently a severe and painful disease that caused - and still causes - economic losses to farmers. No effective treatment has been available. Dermatitis in general has poor prognosis with the need for prolonged treatments. Antibiotic therapy (penicillin, tetracycline, and Trimethoprim-sulphamethoxazole) administered via feed has been attempted, mostly with no effect. If antibiotics (penicillin, amoxicillin, and amoxicillin clavulanate) are administered as injections in the early stage of the disease with a large dosage, clinical signs and morbidity may decrease although relapses are common. When treating tens or hundreds of thousands of semi-wild animals, long term administration of injections is impractical. The housing system also makes it difficult to detect the early signs of the disease thus critically delaying the onset of treatment. Current recommendation is to euthanize the first infected animals, when detected, and then disinfect the cages.

Here, we describe the clinical signs and the gross- and histopathology of the disease in three species. We also describe the major microbiological findings. We found *Arcanobacterium phocae* in all of the diseased animals, which was confirmed both by sequencing and phenotypic characterization. Initially, *A. phocae* was cultured from 50% of the diseased individuals and none of the healthy animals. Notably, all the diseased animals were further confirmed as *A. phocae* positive by rtPCR. Furthermore, necrosis is one of the typical changes that were observed in the diseased fur animals, and it is known that bacteria belonging to the genus *Arcanobacterium* produce toxins with dermonecrotic activity [18]. In some healthy controls, low amounts of *A. phocae* were detected by PCR. These individuals were either healthy mink from an affected farm or finnraccoons, of which half were from a supposedly disease-free farm. Later, the disease was reported to affect animals on this farm. Statistical analysis revealed a significant correlation for all species between FENP and *Arcanobacterium phocae* when detected by PCR, and culturing results gave a strong correlation for mink and foxes.

The samples of mink and finnraccoons presented with chronic inflammation that may have influenced the results. During the study period it was difficult to obtain lesions of the early stages, because the signs and lesions appeared to develop rapidly. Due to the housing system of fur animals, the farmers do not handle the animals individually, which makes it difficult to detect early signs of the disease. Although infection by *A. phocae* may indeed be the primary cause of the disease, it is also possible that the onset of the disease could have been covered by a secondary bacterial infection caused by *A. phocae*. Some of the findings suggest that *A. phocae* can be naturally found on the skin of fur animals, and therefore might not be able to cause the disease alone. Thus, another factor, such as a bite wound, a scratch, a mechanical injury, or another microbial agent, might be required for the disease to proceed.

With histopathology, hyperkeratosis and ulceration of the epidermis were detected, which could be caused by trauma. However, especially in the samples from mink, the inflammation was mainly deep diffuse purulent infection with prominent vasculitis. Vasculitis causes thrombosis, which leads to necrosis of the skin tissue. The pathogenesis of vasculitis generally varies between animal diseases. It is seen in viral diseases such as feline infectious peritonitis, as well as in bacterial infections such as erysipelas in swine. In cats and dogs, similar skin lesions are detected in mycobacterial infections. Most of the infections are due to penetrating injury through skin to the subcutis with soil or dirt contamination. These infections can, with time, become deep-seated and extensive [19]. However, the histopathological pattern of the fur animal samples was atypical to mycobacterial infections and ZN stains did not demonstrate any acid fast bacteria. Parasitic agents, immune-mediated disease and toxins are also known to cause vasculitis and otherwise predispose animals to infection. However, no parasites were detected in this study.

A viral pathogen was considered, since early-onset signs in foxes included serous discharge from the eyes, usually associated with viral infections. Furthermore, with histology, occasional inclusion body -like structures were noted. However, viral cultures from the affected tissue samples did not reveal any viral agents, although this does not exclude a viral cause of the disease. Distemper could manifest as skin changes seen in the mink, and also conjunctivitis in foxes, but, no CDV or antibodies against it were detected in any studied animals. MEV DNA was detected in one healthy fox. Both healthy and diseased foxes and finnraccoons had antibodies against MEV, partly due to vaccinations. All the mink were tested against AMDV, another parvovirus affecting fur animals, with negative results. Furthermore, a screen for herpesviruses using a broadly-reactive PCR protocol also yielded negative results.

Bacterial cultures and subsequent sequencing also revealed a novel *Streptococcus* sp. of probable marine origin. This could reinforce the suggested connection between *A. phocae*-caused pyoderma and the feeding of fur animals with marine mammal byproducts [1]. Alternatively, this novel *Streptococcus* could just be a previously undetected member of the normal flora of the fur animals. The novel *Streptococcus* was not detected as frequently as *A. phocae*, and thus it is not as likely as *A. phocae* to be the main etiological agent. Statistical analysis did show a correlation between FENP and the novel *Streptococcus* sp., and thus indicates its role as a significant cofactor, warranting further studies for its basic characterization. Interestingly the novel *Streptococcus* was found less frequently in foxes than in mink and finnraccoons, which could contribute to the differences in the manifestation of the disease.

This study demonstrated that the bacterium *Arcanobacterium phocae* is associated with the new disease, FENP, seen in fur animals, and suggests that it plays an important role in the pathogenesis of the disease. In further studies, the pathogenicity of *Arcanobacterium phocae* to mink, foxes, and finnraccoons should be investigated by experimental infections to clarify whether Koch's postulates can be fulfilled, i.e. whether it is capable of causing the described disease, or whether other environmental, immunological, or infectious factors are necessary. In conclusion, this study described a new, economically important, and potentially lethal disease of fur animals that severely compromises animal welfare on farms. The study also revealed its likely etiological agent, *A. phocae*, suggesting a potential species shift from marine mammals as the origin of this epidemic.

## Supporting Information

Table S1 **Microbiological results of individual animals in the study.**
(PDF)
