# Supplementary material for: Characterization of a New Epidemic Necrotic Pyoderma in Fur Animals and Its Association with Arcanobacterium phocae Infection
Source: PLoS One. 2014 Oct 10;9(10):e110210. doi: 10.1371/journal.pone.0110210 (PMC4193818; doi:10.1371/journal.pone.0110210)
Supplement: Table S1 — Microbiological results of individual animals in the study. (PDF) [file pone.0110210.s001.pdf]

Table S1. Microbiological results of individual animals in the study.

	farm	Sample nro	*Arcanobacterium phocae*				
			PCR	High bacterial load CT<31	Phocae cultivation	Streptococcus spp. (this study)	Other bacteria
**MINKS**							
DISEASED	A	Pa 835					
	A	Pa 836					
	A	Pa 837					
	A	Pa 838					
	A	Pa 839					
	A	Pa 840					
	A	Pa 841					
	A	Pa 842					
	A	Pa 843					
	A	Pa 844					
	A	Pa 845					
	A	Pa 886					
	A	Pa 887					
	A	Pa 888					
	A	Pa 889					
	A	Pa 890					
	A	Pa 891					
	D	Pa 892					
	D	Pa 893					
	D	Pa 894					
	D	Pa 895					
HEALTHY	C	pa 985					
	C	pa 986					
	C	pa 987					
	C	pa 988					
	C	pa 989					
	C	pa 990					
	C	pa 991					
	C	pa 992					
	C	pa 993					
	C	pa 994					
	C	pa 995					
Healthy animals on a diseased farm	A	Pa 1					
	A	Pa 2					
	D	Pa 3					
	D	Pa 4					
**FINNRACCOONS**							
DISEASED	B	Pa 857					
	B	Pa 858					
	F	pa 914					
	F	Pa 915					
	F	Pa 916					
	F	Pa 917					
	G	Pa 918					
	H	Pa 922					
	H	Pa 923					
	H	Pa 924					
	H	pa 925					
	F	Pa 944					
	F	Pa 945					
	F	Pa 946					
	F	Pa 947					
	F	pa 948	no sample for PCR				
	F	Pa 949					
	F	PA 39					
	F	PA 40					
	K	pa 43					
	K	pa 44					
HEALTHY	A	Pa 876					
	A	Pa 877					
	A	Pa 878					
	A	Pa 879					
	A	Pa 880					
	A	Pa 881					
	A	Pa 907					
	A	Pa 908					
	A	Pa 909					
	A	Pa 910					
	A	pa 911					
**FOXES**							
DISEASED	C	Pa 882					
	C	Pa 883					
	C	Pa 884					
	E	Pa 898					
	E	Pa 899					
	E	Pa 900					
	E	Pa 919					
	E	Pa 920					
	E	Pa 921					
	E	pa 928					
	E	Pa 929					
	E	pa 930					
	E	Pa 931					
	E	Pa 932					
	J	Pa 30					
	J	Pa 31					
	J	Pa 32					
	J	Pa 33					
	J	Pa 34					
HEALTHY	B	pa 953					
	B	pa 954					
	B	pa 955					
	B	pa 956					
	B	pa 957					
	B	pa 958					
	B	pa 959					
	B	pa 960					
	B	pa 961					
	B	pa 962					
	B	pa 963					
	B	pa 964					
